# “Take It One Dilation at a Time”: Caregiver Perspectives of Postoperative Anal Dilations in Pediatric Patients with Colorectal Conditions

**DOI:** 10.3390/bs14050379

**Published:** 2024-04-30

**Authors:** Lea A. Wehrli, Merlin Ariefdjohan, Jill Ketzer, Kristina Matkins, Luis De la Torre, Andrea Bischoff, Laura Judd-Glossy

**Affiliations:** 1 International Center for Colorectal and Urogenital Care, Children’s Hospital Colorado, Aurora, CO 80045, USA; lea.wehrli@wvu.hsc.edu (L.A.W.); jill.ketzer@childrenscolorado.org (J.K.); kristina.matkins@childrenscolorado.org (K.M.); luis.delatorre@childrenscolorado.org (L.D.l.T.); andrea.bischoff@childrenscolorado.org (A.B.); 2 Child and Adolescent Mental Health Division, Department of Psychiatry, University of Colorado Anschutz Medical Campus, Aurora, CO 80045, USA; merlin.ariefdjohan@cuanschutz.edu; 3 Pediatric Mental Health Institute, Children’s Hospital Colorado, Aurora, CO 80045, USA

**Keywords:** anal dilations, anorectal malformation, Hirschsprung disease, caregiver experiences, caregiver perspectives, psychosocial support

## Abstract

Background: Postoperative anal dilations (PAD) are the standard of care for patients after a posterior sagittal anorectoplasty (PSARP) for anorectal malformation (ARM) or a transanal pull-through (TP) procedure for Hirschsprung disease (HD). This study assessed the psychosocial impact of PAD among caregivers of children with ARM or HD, which may inform postoperative care strategies. Methods: Caregivers of patients with ARM and HD who underwent PSARP or TP within five years participated in the online survey. Questions included demographics, patient and caregiver experiences with PAD, and baseline psychosocial functioning. Quantitative results were reported descriptively, while qualitative responses were summarized as major themes. Results: The survey indicated a response rate of 26% caregivers, with most being female (91%) and biological mothers (85%). Patients were mostly male (65%), born with ARM (74%), and were five months old on average when PAD began. Caregivers reported that during PAD, children experienced distress (56%), pain (44%), and fear (41%), while a third noted no negative reactions. Over time, their child’s ability to cope with PAD got easier (38%) or stayed the same (41%). Caregivers reported worry/anxiety (88%), guilt (71%), stress (62%), and frustration (35%), noting that additional coping strategies to manage the emotional and logistical challenges of daily PAD would be helpful. Conclusion: Although PAD is necessary, it can be highly stressful for the patients and their caregivers. Key findings emphasized the need for additional coping strategies and highlighted the importance of integrating psychosocial support into the postoperative care regimen.

## 1. Introduction

Postoperative anal dilations (PAD) are the standard of care in patients who undergo a posterior sagittal anorectoplasty (PSARP) for anorectal malformation (ARM) or a transanal pull-through (TP) procedure for Hirschsprung disease (HD) [1,2,3]. In 1982, Pena and DeVries recommended the PAD regimen following the PSARP procedure to prevent stricture formation of the neoanus [2]. Similar recommendations have been made for patients with HD who undergo a TP [3]. However, there is great variability in the frequency and modality of dilations worldwide, ranging from twice daily by caregivers to once weekly by surgeons [3]. In addition, the necessity and effectiveness of routine dilations have been debated regarding stricture prevention in PSARP and TP procedures [4,5,6]. For example, in one study, patients with HD, who completed dilations, had delayed onset of strictures, whereas in patients without dilations, strictures occurred in the early postoperative course, and no difference was found in stricture rates between those two groups [5]. Similarly, another study found no difference in the postoperative complication rate in patients with ARM [4]. In contrast, a recent retrospective study of patients with ARM who underwent a PSARP and followed a strict dilation protocol postoperatively for 9 months found that no strictures occurred [6].

Regardless of the postoperative protocol of anal dilations, it is important to consider the clinical impact on the patient, the patient–caregiver relationship, and the future impact on the patient’s psychosocial functioning. Diseth and colleagues completed numerous studies in the 1990s [7,8,9,10], which raised concern that patients who underwent PAD might experience long-term psychological sequelae or be at an increased risk for a psychiatric diagnosis [10]. For example, in one study, adolescent patients who completed daily dilations in early childhood (up to 4 years old) for several years had higher rates of mental health concerns and psychosocial issues compared to adolescent patients with HD who did not undergo dilations [9]. However, there have not been any recent studies to replicate these findings or evaluate the impact of PAD on the patient and their caregiver.

Thus, this study aimed to assess the patient and caregiver’s experience of performing PAD, evaluate the psychosocial impact of this postoperative medical care, and consider ways to better support patients who require anal dilations and their caregivers.

## 2. Materials and Methods

### 2.1. Survey and Study Participants

A brief survey was developed by the study’s authors to evaluate caregivers’ experience of performing anal dilations on their children following surgery for correcting colorectal disorders. Participants were parents or guardians who have performed PAD on their child before or after they had surgery at the study site, and thus were registered in the clinic registry. Eligible caregivers were invited to participate in the survey via email, which was subsequently administered using the Research Electronic Data Capture (REDCap) platform, hosted at the University of Colorado Anschutz Medical Campus [11]. Personal identifiers including IP addresses were not collected during the survey to preserve caregivers’ anonymity. The survey was administered for approximately three weeks from February to March 2023.

Briefly, the 54-item survey gathered information related to the basic demographic profile of the caregivers, the basic clinical profile of their child, sentiments related to their experience in performing PAD, sentiments related to their child’s experience in having anal dilations, their experience with providers, and other perspectives to share with other parents dealing with a similar situation. Responses were assessed through a combination of multiple-choice questions, rating personal sentiments on the Likert scale, and sharing thoughts as open-ended responses. The survey questionnaire can be viewed via this link: https://drive.google.com/file/d/1txjoG6tYrV_m46AYRvtUaBR2OY5aGoTs/view?usp=drive_link (accessed on 1 February 2023).

This study received the approval of the Colorado Multiple Institutional Review Board (COMIRB), which provides regulatory oversight for human subject research at the study site (Protocol 22-1716).

### 2.2. Data Analyses

Quantitative data were analyzed using descriptive statistics (e.g., count, percent frequency) and summarized as pertinent trends when applicable (SAS Studio, SAS Inc., Cary, NJ, USA). A thematic analysis framework was used to organize and identify major themes from open-ended responses to respective survey questions. These qualitative responses were first independently reviewed by the authors (LJG, MA) to minimize subjectivity, then discussed and grouped together by the authors as major themes supported by pertinent quotes through inductive reasoning. This qualitative research methodology is largely based on the consensual qualitative research (CQR) process [12].

## 3. Results

The online survey was disseminated via email to a total of 138 caregivers in the clinical registry. Out of these, 7 emails were returned undelivered, ineligible, or requested to be removed from the list, to provide a total number of eligible respondents of 131. At the end of the study period, 34 caregivers completed the survey, which reflected a 26% response rate.

### 3.1. Basic Demographic Profile of Caregivers and Clinical Profile of Their Children

More detailed information is outlined in Table 1. Briefly, most parent/caregivers (n = 34) were female (91.2%) and identified as the biological mother of the child (85.3%). Caregivers’ children were mostly male (64.7%), with a median age of 6 months (range from 2 weeks to 5 years of age). Generally, the top three colorectal disorders reported were recto-perineal fistula (23.5%), imperforate anus without fistula (20.6%), and HD (20.6%). Approximately 70.6% of caregivers mentioned that their child did not require additional surgery to the anus after the main operation (e.g., PSARP, PSARVUP, TP), four (11.8%) of those who required surgery needed it for rectal prolapse, and three (8.8%) were operated on elsewhere initially but had to re-do the PSARP for stricture.

### 3.2. Caregivers’ Logistical Experience in Performing Anal Dilations on Their Child

Caregivers were asked to indicate when their child first had an anal dilation. A wide age range was mentioned ranging from approximately 1 week old to 19 months of age. Further, most caregivers noted that although they performed anal dilations on the child together with their partner (76.5%), they performed the procedure on their own more than 50% of the time (82.4%). Several caregivers also mentioned receiving help from “others” such as family members and an in-home nurse. In terms of the timing of when they began anal dilations, 79.4% of caregivers indicated not performing the procedure prior to surgical repair, but 97.1% mentioned performing this on their child postoperatively. Approximately 23.5% of caregivers conducted the procedure for less than 3 months, 41.2% for 3 to 6 months, and 32.4% for longer than 6 months. These results are inconsistent with the anal dilation protocol at our institution, which is approximately nine months postoperatively. These results are outlined in Table 1.

### 3.3. Caregivers’ Personal Sentiments about Their Child’s Experience in Receiving Anal Dilations and Their Experience in Performing Anal Dilations on Their Child

Caregivers (n = 34) reported that their children experienced distress, pain, and/or fear of dilations (as rated by 52.9%, 44.1%, and 41.2% of respondents, respectively; Table 2). However, 32.4% indicated that their child did not experience any negative reactions during the procedure. There were also caregivers (14.7%) who mentioned observing their child having sentiments other than distress, pain, and fear during dilations. They mentioned “*Panic. Long term trauma*”. (child’s age at dilations: 2 months); “*As his age increased, he had more fear and distress, but initially he did not have any reaction*”. (6 months); “*Very little negative reaction, sometimes some minor discomfort from the pressure*”. (as newborn then after PSARP performed at approximately 2 months of age); “*I wouldn’t say he was distressed, but he didn’t enjoy this at the beginning of the process. It was normalized as we progressed through the dilation schedule*”. (5 years old); and “*My baby seems to be quiet and looking—but he didn’t seem to be in any pain. I’m concerned if he will remember and if it can affect him in adulthood*”. (4 to 8 months of age).

On the other hand, caregivers reported feeling worry/anxiety, guilt, stress, and/or frustration towards performing dilations on their child (as rated by 88.2%, 70.6%, 61.8%, and 35.3% of respondents, respectively; Table 2). Among those who shared that they experienced sentiments other than those listed here, they mentioned: “*I’m ok with it, because it’s something that has to be done for her health and I’ll do what I have to make sure she is safe and healthy*”; “*Depression/ deep sadness and distress*”; “*It was very stressful at first, but over time it became much less so. More just a regular routine.*”; and “*I didn’t want to see/hear cry, but it went so quick that we got through it*”.

Approximately half of the caregivers indicated that performing dilations became easier over time, but 10% of respondents expressed that it stayed the same, and 20.6% stated that it became harder (Table 2). However, many caregivers reported that it mostly became easier (38.2%) or stayed the same for their child (41.2%). One respondent further shared that “*It got much easier and less stressful rather quickly…Our child coped well and never seemed very bothered by them*”. Notably, many caregivers indicated having moderate worry/anxiety on a daily basis (50.0%), with the majority expressing being more worried/anxious when performing dilations (61.8%) (Table 2). However, those that reported less worry/being anxious shared the following: “*I was more worried and anxious initially, but that declined pretty quickly and so it became a routine like changing a dirty diaper.*”; and “*We were in the throws of our first 6 months of adoption, as brand new parents. Throw surgery on top of that, so it’s difficult to be able to isolate exactly what changes dilation produced in this mix. I would say initially it caused some concern, and then as time progressed, this concern diminished*”. Additionally, most caregivers indicated being more stressed than usual when performing dilations (55.9%), and feeling more guilt than usual (55.9%), but did not notice any changes in overall frustration (55.9%). One respondent shared that “…*it was more stress initially, [but] it declined quickly and became more routine and not stressful*”, and “*it was frustrating at first, but that quickly dissipated*”.

Notably, most caregivers indicated “no” (82.4%) when asked “If you were given the option, would you have preferred to not complete dilations on your child and instead take the risk of your child needing another operation under anesthesia?” (Table 2). Several shared the following statements: “…*she has been through enough surgeries and if I can prevent a surgery by doing the dilations, that’s what I’ll do and did do*”; “…*would have been nice, but I understand the necessity of it*”; and “…*definitely would rather do dilations and not have my son under anesthesia*”. However, there were also caregivers who indicated being unsure (8.8%) and cited reasons as “*Dilators have been challenging and our daughter has a lot of anxiety, pain, and fear…If they were able to do a procedure to eliminate the need for dilators and the recovery was quick, we likely would be interested in learning more. If it required a multi-day hospital stay, we likely would not*”.

Considering the burden of performing anal dilations on their child, several caregivers expressed that they wished they were better prepared emotionally and in terms of logistics (“*The stress that it causes you. It doesn’t get any easier doing and sometimes it will mentally wear you down and make you feel like a horrible person*.”; “*What the duration was. How to properly administer it. Signs it wasn’t working*.”) (Table 3). However, there were also others who mentioned “*I don’t feel there was anything that would have made a difference*” and that they were “*already well informed*” (Table 3). Nonetheless, caregivers found the support from spouses/family and medical providers when performing dilations to be impactful (Table 3). Further, several caregivers shared child- and parent-specific strategies to facilitate the anal dilations process. These included keeping the child distracted by watching video or singing songs during the procedure, implementing physical treatment measures (e.g., “…*use lots of lube*”), mentally being prepared that the procedure was needed for the health of the child, and performing self-calming techniques prior to performing the procedure (e.g., “*take a deep breath*”; “*take it one dilation at a time*”, among others) (Table 3).

### 3.4. Social Support for Caregivers

While necessary, there is no doubt that PAD is an invasive procedure and can contribute substantially to the psychological burden of the person completing the procedure. Respondents mentioned that performing anal dilations on their child was anxiety-provoking and could be “*terrifying*”, and that “*dilations were the most difficult part of motherhood for me thus far*” (Table 3). Due to this, several caregivers strongly advocated for finding social and mental health support for themselves, which can include consulting with a therapist, maintaining active communication with their medical providers, participating in a peer support group, and talking to others experiencing a similar situation on a 1:1 basis (Table 3).

### 3.5. Caregivers’ Experience with Their Providers

All caregivers mentioned that their providers explained the purpose of PAD, including providing information that their child might need a reoperation if dilations were not completed postoperatively (Table 2). Further, the majority (97.1%) indicated receiving sufficient support from their providers when they had just started performing anal dilations (Table 2). One respondent further mentioned that “…[being given] *more information helps us feel better-equipped to tackle new experiences, such as dilation*”. Overall, there were mixed sentiments when respondents were asked if they wished their provider had done anything differently to prepare them for the dilations. Some caregivers indicated they received sufficient support (“*There was nothing more that could* [have] *been provided or told*”.), while others needed more support (“…*I wish there was more support and or follow up for how hard it is to go through that with your child*…”) (Table 3). They also cited provider-specific qualities that promoted feelings of being satisfied with the care, which were mostly attributed to providers’ confidence, skills, and knowledge during clinical consultation (Table 3). Two caregivers who initially received care at a different hospital and later transferred care to our hospital, reported very different experiences with PAD. Notably, two caregivers highlighted the discrepancy in the quality of care since they experienced two providers at two different locations (Table 3).

## 4. Discussion

Anal dilations are performed worldwide as postoperative care for patients who undergo a PSARP or TP procedure. While there is a common understanding that anal dilations can be stressful for the patient [3,5], there are limited studies that assess the caregivers’ perspective on completing them. In our retrospective study, many caregivers shared that performing the PAD regimen was distressing and anxiety-provoking for them, and perceived that it was difficult for their children as well. Despite these challenging experiences, most caregivers noted that they preferred to complete these medical tasks, rather than risk their child needing another operation or going under anesthesia as part of the invasive procedure.

Thus, according to the majority of our respondents, the question is less about *whether* to complete anal dilations, but more about *how* to support patients and families during this process. Caregivers who had completed PAD shared many recommendations about the support that could have made this process easier for them, including both logistic details (e.g., when they could stop completing dilations) as well as emotional support (e.g., acknowledging that caregivers might feel guilt or other negative emotions when starting these procedures). In addition, many respondents discussed the need for more specific and tangible strategies to help their children cope with the PAD regimen. Thus, clinical care teams need to be cognizant of this challenge and find ways to integrate psychosocial support for caregivers to directly address these burdens.

Psychosocial support is a necessary and key component to providing excellent medical care to patients with colorectal conditions and their families [13]. Psychologists and social workers are well-equipped to directly apply the recommendations found in this study by meeting with patients and families when they are starting dilations to discuss the emotional components for caregivers and patients alike. In addition, these psychosocial providers can also use their expertise in child development to help caregivers brainstorm developmentally appropriate coping skills for their children during the medical care itself. The opportunity to connect with other families of children with colorectal conditions can also be extremely helpful for caregivers [14]. In particular, online support groups have been shown to provide families with a unique source for information, support, and connection [15].

This study has several limitations, primarily as reflected in the small sample size and low response rate. Thus, it is difficult to fully generalize these results to the broader experience of patients who require PAD and their caregivers, as well as to extrapolate the results to other medical conditions. Because this study aimed to be a singular descriptive examination and lacked established questionnaires tailored to its subject matter, the authors created their own survey instruments. Although these instruments were not formally validated, they were crafted based on the authors’ collective expertise, including backgrounds as a clinical psychologist, pediatric surgical fellow, clinical social worker, and director of a pediatric colorectal surgery center. Additionally, they drew upon insights gleaned from literature reviews, participation in relevant professional conferences and meetings (such as Pull-thru Network and the American Pediatric Surgery Association), and individual interactions with parents in clinical settings, particularly at pediatric colorectal centers. The retrospective nature of the study may also impact the results, particularly since some caregivers completed dilations 3 to 5 years ago, which may contribute to recall bias. Similarly, many caregivers noted that they stopped performing PAD before the full nine-month protocol; however, the survey did not gather information about the rationale for not completing the recommended protocol. Additionally, since our participant population is unique, this further limited the size of the participant pool that was available to us. Finally, the study did not examine the individual clinical course of patients (e.g., complications during dilations, prolapse, bleeding), which could directly impact the experience of the patient and their caregiver.

Despite the study limitations and considering the limited extent of the current literature in this area, key findings from this study remain valuable in highlighting challenges that caregivers faced when caring for children with colorectal conditions. Performing a PAD regimen can be highly stressful for both patients and their caregivers. This emphasizes the critical need for colorectal providers to support families throughout the dilation regimen, including offering strategies to promote positive coping in children and caregivers. These psychosocial supports could include the provision of direct clinical care to caregivers by psychologists and social workers, promoting the use of caregiver support groups, and helping families advocate for the needs of their children and themselves with their providers. These strategies will allow caregivers to be more adept in caring for their children and for themselves throughout the process. Overall, psychosocial support for patients and families should receive the same level of priority and attention as the patient’s medical care. The clinical care team could consider incorporating these strategies to elevate the quality of patient care in this field.

## 5. Conclusions

Anal dilations are performed postoperatively in patients who undergo PSARP or TP for colorectal conditions such as ARM or HD. Despite caregivers’ awareness of the medical necessity for performing PAD, this regimen is perceived as highly stressful for both patients and caregivers. This study highlights the need for colorectal providers to integrate psychosocial support and offer coping strategies to help with managing the emotional and logistical challenges of daily dilations, to complement the physical care given to the patient. Further, considering the limited availability of information in the existing literature, longitudinal studies are warranted to better assess the impact of the postoperative care regimen (such as PAD) on patients’ and caregivers’ long-term psychosocial functioning.

## Figures and Tables

**Table 1:** Basic demographic and clinical profile of parents/guardians and their children. All results are presented as % (n).

Variable	Child Age at Initial Dilation (<6 Months; n = 11)	Child Age at Initial Dilation (6 to 12 Months; n = 16)	Child Age at Initial Dilation (>12 Months; n = 7)	All Data (N = 34)
**How do you identify as a caregiver:**				
Biological mother	81.8 (9)	100.0 (16)	57.1 (4)	85.3 (29)
Biological father	18.2 (2)	0 (0)	14.3 (1)	8.8 (3)
Adoptive mother	0 (0)	0 (0)	28.6 (2)	5.9 (2)
Adoptive father	0 (0)	0 (0)	0 (0)	0 (0)
Other family member	0 (0)	0 (0)	0 (0)	0 (0)
**What gender do you identify with?**				
Female	81.8 (9)	100.0 (16)	85.7 (6)	91.2 (31)
Male	18.2 (2)	0 (0)	14.3 (1)	8.8 (3)
Nonbinary	0 (0)	0 (0)	0 (0)	0 (0)
**What gender is your child?**				
Female	18.2 (2)	56.3 (9)	14.3 (1)	35.3 (12)
Male	81.8 (9)	43.8 (7)	85.7 (6)	64.7 (22)
Nonbinary	0 (0)	0 (0)	0 (0)	0 (0)
**What is your child’s diagnosis?**				
Recto-perineal fistula	45.5 (5)	18.8 (3)	0 (0)	23.5 (8)
Recto-vestibular fistula	2.9 (1)	0 (0)	0 (0)	2.9 (1)
Imperforate anus without fistula	9.1 (1)	25.0 (4)	28.6 (2)	20.6 (7)
Recto-urethral bulbar fistula	0 (0)	6.3 (1)	0 (0)	2.9 (1)
Recto-urethral prostatic fistula	9.1 (1)	6.3 (1)	14.3 (1)	8.8 (3)
Recto-bladderneck fistula	9.1 (1)	0 (0)	0 (0)	2.9 (1)
Cloaca	0 (0)	18.8 (3)	14.3 (1)	11.8 (4)
Hirschsprung	9.1 (1)	18.8 (3)	42.9 (3)	20.6 (7)
I don’t know	9.1 (1)	6.3 (1)	0 (0)	5.9 (2)
**After the main operation to create the anus (PSARP, PSARVUP, Pullthrough), did your child require additional surgery of the anus?**				
Yes	45.5 (5)	0 (0)	71.4 (5)	29.4 (10)
No	54.6 (6)	100.0 (16)	28.6 (2)	70.6 (24)
**Why did your child need an** **additional surgery?**				
Due to stricture (narrowing)	40.0 (2)	0 (0)	20.0 (1)	30.0 (3)
Due to prolapse (red tissue coming out)	40.0 (2)	0 (0)	40.0 (2)	40.0 (4)
I don’t know	0 (0)	0 (0)	0 (0)	0 (0)
Other reason	20.0 (1)	0 (0)	40.0 (2)	30.0 (3)
**When did your child begin dilations?**				
2012	0 (0)	0 (0)	14.3 (1)	2.9 (1)
2015	9.1 (1)	0 (0)	0 (0)	2.9 (1)
2016	0 (0)	0 (0)	0 (0)	0 (0)
2017	9.1 (1)	0 (0)	0 (0)	2.9 (1)
2018	9.1 (1)	12.5 (2)	0 (0)	8.8 (3)
2019	36.4 (4)	25.0 (4)	57.1 (4)	35.3 (12)
2020	18.2 (2)	12.5 (2)	14.3 (1)	14.7 (5)
2021	9.1 (1)	37.5 (6)	14.3 (1)	23.5 (8)
2022	9.1 (1)	12.5 (2)	0 (0)	8.8 (3)
**Who, at any time, did a dilation on your child? (please select all** **relevant answers)**				
Me (by myself)	36.4 (4)	68.8 (11)	28.6 (2)	50.0 (17)
My partner (by himself/herself)	18.2 (2)	25.0 (4)	28.6 (2)	23.5 (8)
Me and my partner together	81.8 (9)	62.5 (10)	100.0 (7)	76.5 (26)
Someone else	9.1 (1)	6.3 (1)	0 (0)	5.9 (2)
**Who did the majority (more than 50%) of the dilations?**				
Me (by myself)	81.8 (9)	87.5 (14)	71.4 (5)	82.4 (28)
My partner (by himself/herself)	9.1 (1)	0 (0)	14.3 (1)	5.9 (2)
Me and my partner together	9.1 (1)	6.3 (1)	14.3 (1)	8.8 (3)
Someone else	0 (0)	6.3 (1)	0 (0)	2.9 (1)
**Did you do anal dilations** **before surgery?**				
Yes	18.2 (2)	12.5 (2)	28.6 (2)	17.7 (6)
No	81.8 (9)	87.5 (14)	57.1 (4)	79.4 (27)
Other	0 (0)	0 (0)	14.3 (1)	2.9 (1)
**Did you do anal dilations** **after surgery?**				
Yes	100.0 (11)	93.8 (15)	100.0 (7)	97.1 (33)
No	0 (0)	6.3 (1)	0 (0)	2.9 (1)
**How long did you do anal dilations?**				
Less than 3 months	36.4 (4)	25.0 (4)	0 (0)	23.5 (8)
3–6 months	18.2 (2)	43.8 (7)	71.4 (5)	41.2 (14)
More than 6 months	36.4 (3)	31.3 (5)	28.6 (2)	32.4 (11)
Other	9.1 (1)	0 (0)	0 (0)	2.9 (1)

**Table 2:** Parents/guardians’ perceived sentiments related to performing anal dilations on their child, and their general experience with providers. All results are presented as % (n).

Variable	Child Age at Initial Dilation (<6 Months; n = 11)	Child Age at Initial Dilation (6 to 12 Months; n = 16)	Child Age at Initial Dilation (>12 Months; n = 7)	All Data (N = 34)
**My child experienced the following reactions to dilations (select all** **that apply)**				
Fear	36.4 (4)	25.0 (4)	85.7 (6)	41.2 (14)
Distress	45.5 (5)	43.8 (7)	85.7 (6)	52.9 (18)
Pain	45.5 (5)	31.3 (5)	71.4 (5)	44.1 (15)
My child did not experience any negative reactions	36.4 (4)	43.8 (7)	100.0 (7)	32.4 (11)
Other	27.3 (3)	6.3 (1)	14.3 (1)	14.7 (5)
**I experienced the following emotions when doing dilations on my child (select all that apply)**				
Worry/Anxiety	90.9 (10)	87.5 (4)	85.7 (6)	88.2 (30)
Guilt	63.6 (7)	68.8 (11)	85.7 (6)	70.6 (24)
Stress	63.6 (7)	50.0 (8)	85.7 (6)	61.8 (21)
Frustration	45.5 (5)	18.8 (3)	57.1 (4)	35.3 (12)
Other	27.3 (3)	12.5 (2)	14.3 (1)	17.7 (6)
**Across the time that you did dilations, how did your ability to do dilations change over time?**				
It got easier	36.4 (4)	62.5 (10)	42.9 (3)	50.0 (17)
It stayed the same	36.4 (4)	25.0 (4)	28.6 (2)	29.4 (10)
It got harder	27.3 (3)	12.5 (2)	28.6 (2)	20.6 (7)
Other	0 (0)	0 (0)	0 (0)	0 (0)
**Across the time that your child received dilations, how did their ability to cope with dilations change over time?**				
It got easier	27.3 (3)	37.5 (6)	57.1 (4)	38.2 (13)
It stayed the same	45.5 (5)	50.0 (8)	14.3 (1)	41.2 (14)
It got harder	18.2 (2)	12.5 (2)	28.6 (2)	17.7 (6)
Other	9.1 (1)	0 (0)	0 (0)	2.9 (1)
**In general, how would you rate your worry/anxiety on a day to day basis?**				
I typically have little worry/anxiety	18.2 (2)	25.0 (4)	71.4 (5)	32.4 (11)
I typically have moderate worry/anxiety	54.6 (6)	56.3 (9)	28.6 (2)	50.0 (17)
I typically have high worry/anxiety	27.3 (3)	18.8 (3)	0 (0)	17.7 (6)
Other	0 (0)	0 (0)	0 (0)	0 (0)
**Did you notice any changes in your overall worry/anxiety when you were doing dilations?**				
I was less worried/anxious overall than usual for me	0 (0)	0 (0)	0 (0)	0 (0)
I did not notice any changes in my overall worry/anxiety	18.2 (2)	56.3 (9)	0 (0)	32.4 (11)
I was more worried/anxious overall than usual for me	72.7 (8)	43.8 (3)	85.7 (6)	61.8 (21)
Other	9.1 (1)	0 (0)	14.3 (1)	5.9 (2)
**Did you notice any changes in your overall stress when you were doing dilations?**				
I was less stressed than usual for me	9.1 (1)	0 (0)	0 (0)	2.9 (1)
I did not notice any changes in my overall stress level	36.4 (4)	50.0 (8)	14.3 (1)	38.2 (13)
I was more stressed than usual for me	45.5 (5)	50.0 (8)	85.7 (6)	55.9 (19)
Other	9.1 (1)	0 (0)	0 (0)	2.9 (1)
**Did you notice any changes in your overall feelings of guilt when you were doing dilations?**				
I felt less guilty than usual for me	0 (0)	0 (0)	0 (0)	0 (0)
I did not notice any changes in my feelings of guilt	45.5 (2)	56.3 (9)	14.3 (1)	44.1 (15)
I felt more guilty than usual for me	54.6 (6)	43.8 (7)	85.7 (6)	55.9 (19)
Other	0 (0)	0 (0)	0 (0)	0 (0)
**Did you notice any changes in your overall frustration when you were doing dilations?**				
I was less frustrated than usual for me	0 (0)	0 (0)	14.3 (1)	2.9 (1)
I did not notice any changes in my overall frustration	54.6 (6)	75.0 (12)	14.3 (1)	55.9 (19)
I was more frustrated than usual for me	36.4 (4)	25.0 (4)	71.4 (5)	38.2 (13)
Other	9.1 (1)	0 (0)	0 (0)	2.9 (1)
**Did a provider explain the purpose of dilations (e.g., that your child might need a reoperation without dilations)?**				
Yes	100.0 (11)	100.0 (16)	100.0 (7)	100.0 (34)
No	0 (0)	0 (0)	0 (0)	0 (0)
**How would you rate the experience you had with your provider about dilations?**				
My provider gave me enough support in starting dilations	100.0 (11)	100.0 (11)	85.7 (6)	97.1 (33)
My provider did not give me enough support in starting dilations	0 (0)	0 (0)	0 (0)	0 (0)
Other	0 (0)	0 (0)	14.3 (1)	2.9 (1)
**If you were given the option, would you have preferred to not complete dilations on your child and instead take the risk of your child needing another operation under anesthesia?**				
Yes	9.1 (1)	0 (0)	28.6 (2)	8.8 (3)
No	81.8 (9)	93.8 (15)	57.1 (4)	82.4 (28)
Other	9.1 (1)	6.3 (1)	14.3 (1)	8.8 (3)

**Table 3:** Major themes emerging from the respective open-ended questions in the survey, and pertinent quotes from respondents.

**Question: “What do you wish your provider would have done differently when you were starting dilations?”**	
**Themes**	**Quotes**
None or nothing more needed	- “*We were given all the right information*”. - “*There was nothing more that could (have) been provided or told*”. - “*I felt heard and supported*”.
Need more resources, support, and/or advice	- “*Suggestions on how to soothe the child during dilations. Tips on getting the child to cooperate according to their age group*”. - “*I wish there was more support and or follow up for how hard it is to go through that with your child. Our provider gave us the information he needed to for us to make the decisions but doing it was completely heart wrenching*”. - “*It would be nice to know about approximately how deep to go. If we had a marking that said not past this line, that would have reduced a lot of anxiety*”.
Provider-specific qualities that influence satisfaction to care	- “[My provider] *did an excellent job explaining why they needed to be done and the benefits of doing them as well as the possible consequences for not doing them*”. - “*My provider was very supportive. We were inpatient in the NICU at the time so we had a lot of support. I preferred to do the dilations myself*”. - “[My provider] *made it seem so easy and non-invasive, and that’s the approach we took with it*”.
Discrepancy in experience with providers	- “(At the first institution)—[I wish they had given us] *A clear plan of action and listen to us.* (At the second institution)- *nothing they were good*”. - “*I wish we would have had a knowledgeable provider closer to home…To my knowledge*, [my city] *did not and still does not have a surgeon or doctor comfortable with these conditions*”.
**Question: “What do you wish that you knew before starting dilations?”**	
**Themes**	**Quotes**
Emotional preparation expectation/burden	- “*The stress that it causes you. It doesn’t get any easier doing and sometime it will mentally wear you down and make you feel like a horrible person*”. - “*How emotionally taxing it was going to be*”. - “*I wish I would of known the guilt I would feel having to do it to my child. although I knew it was for her best interest and it had to be done so she could heal properly. It is never easy for a parent to do that. No matter how prepared you are*”. - “*I wish I had more confidence that my child wouldn’t mind them*”. - “*How hard mentally and physically it would be for my child*”.
Physical preparation/Logistics	- “*What the duration was. How to properly administer it. Signs it wasn’t working*”. - “*That we could stop eventually. I was unaware that we could stop once we reached our size goal and frequency*”. - “*A little bit more around the reasoning why*”. - “*Just a more gradual, sensitive explanation and demonstration*”.
Nothing: already well-informed	- “*I wouldn’t change anything about the knowledge given to me before dilations*”. - “*I don’t feel there was anything that would have made a difference*”. - “*Nothing I was well informed*”.
Other	- “*I learned as I went along. I never knew this existed until I had my daughter so I’m learning as I go day by day*”. - “*That it would be okay*”. - “*The child at that age doesn’t tend to mind it. I’m sure it’s a different experience when the child is older*”.
**Question: “Who and/or what helped you the most in doing dilations?”**	
**Themes**	**Quotes**
On my own	- “*I did them myself*”. - “*Myself: knowing that I had to and it benefited my child*”.
Spouse and family	- “*Family support*”. - “*Having a partner/caregiver to trade off doing dilations with. It’s emotionally taxing to be the sole person doing dilators each day*”. - “*My fiancé was very supportive in helping by comforting our son as well as myself. He would sometimes have to help hold him while I did it or vice versa*” - “*Support from my husband and realizing my son began to be unaffected by the dilations*”.
Medical care team/provider	- “*Provider support*”. - “*Clear instruction and in office demonstration*”. - “*Staying in touch with the colorectal nurses on our experiences, as we were dilating*”. - “*Having medical/nursing experience prior to beginning dilations helped the most*”.
Child-specific strategies	- “*Allowing him to watch Youtube nursery rhyme videos*”. - “*A schedule, lubrication and a calm environment*”. - “*Singing songs to my child, distracting them with a toy*”. - “*Tucking arms into onesie. Singing a song to distract*”. - “*Keeping my son distracted while doing them made them easier*”.
Parent coping strategies—cognitive	- “*Knowing that it was helping my child and their future depended on it. I wanted as few complications as possible and for my child to have as much of a normal life as possible*”. - “*Knowing we had a timeline...we wouldn’t have to do it forever*”. - “*Just knowing we were trying to prevent additional surgery*”. - “*That it doesn’t hurt, that it’s a little pressure but not painful to the baby*”.
Parent coping strategies—physical	- “*Practice! With just a little bit of time, it became very routine and not stressful*”. - “*Breathing*”.
Other	- “*Peer support*”. - “*I quit doing them*”.
**Question: “What advice would you give to other caregivers starting dilations?”**	
**Themes**	**Quotes**
Techniques/physical strategies	- “*Having a routine and set up of all the necessary equipment makes it easier for everyone*”. - “*Do them at a time that is least stressful for yourself and your child*”. - “*…Use lots of lube*”.
Caregiver coping strategies	- “*Take a deep breath. This is going to hard but you will all get through it*”. - “*Just know it’s to help your child for their future even though it is hard to watch them go through*”. - “*You can do it. It gets easier and turns into no big deal*”. - “*Dilations shouldn’t be a scary thing. You are increasing the likelihood of proper healing and lessening the chance for another anesthetic experience…Give those babies lots of love!*” - “*To take it one dilation at a time. while it may not get easier in the guilt department,* [you’re] *doing exactly what it is supposed to do and you are doing the best thing possible for your child, and that is the most important part of the whole thing.*”
Caregiver social support	- “*I would just endorse that parents needs to appropriately take care of their mental health during that time*”. - “*Know it is going to be awful. Get a therapist. Get a support group. Trust your gut and be a strong advocate for your child. You know your child best*”. - “*Use your resources and help of others*”. - “*Have a support person you can talk to when things get stressful*”.
Patient coping strategies	- “*Distraction is very useful when doing dilations in a toddler. We use electronics and let him watch tv to keep him occupied*”. - “*Communicate with your child when you are doing dilators. We give her her paci, lovey, and then tell her what is going on. We only give her these items on the changing table when she is getting dilators*”. - “*Have on hand great diversion tactics, for the initial dilation. Discuss beforehand, what dilation is and why your child requires it plus what it might feel like*”. - “*The process is different for each situation. As a caregiver, staying calm and talking in a soothing tone to your child can help them stay calm as well*”.
**Question: “Is there anything else you feel we should know about your experience in performing dilations?”**	
**Themes**	**Quotes**
Importance of social support	- “*Having someone to talk to when you don’t have family resources is important. It is not the most positive experience to have as a parent and those without social support should have a resource to help with their mental health. Depending on age of the patient they may need some type of resource as well. Thank you!!!*” - “*If a parent is prescribed dilation for their child, they need mental health support during and after the length of the procedure*”. - “***[name removed to preserve anonymity] and her team is amazing. We have felt so supported throughout this entire process and feel so grateful that our daughter is their patient. My answers above are just to provide context in our experience and hopefully help an A++ program get even better”.
Challenging, but necessary	- “*Being fastidious about sticking to the regime is paramount. If we’re going to do dilations, doing it well, so we know we couldn’t have done anything else to avoid stricture, was worth the ‘inconvenience’ to us*”. - “*I’d rather do the dilations than face another surgery. Of course, we didn’t look forward to this part of our day, but it only takes about a minute, and with all the other things we do medically, this is just another thing, and we got through it. I would do it again if we had to*”.
Anxiety-provoking	- “*I think providers should understand that the process of sticking anything up your child’s anus will come with anxiety and fear of doing it wrong*”. - “*I understand the medical necessity for it but I wish there was a way it wasn’t so painful and uncomfortable. It was an awful experience to have to do that to my infant son and I experienced first-hand how he felt having it done to him*”. - “*Dilations were the most difficult part of motherhood for me thus far*”. - “*Terrifying*”.

## Data Availability

The datasets presented in this article are not readily available because the approved research protocol allows authors only to share aggregate data (summarized as Table 1, Table 2 and Table 3 in the manuscript) and states that authors do not have explicit consent from participants to share individual responses. Questions about the datasets can be directed to the corresponding author at laura.judd-glossy@childrenscolorado.org.

## Abbreviations

ARM	Anorectal Malformation
COMIRB	Colorado Multiple Institutional Review Board
CQR	Consensual Qualitative Research
HD	Hirschsprung Disease
PAD	Postoperative Anal Dilations
PSARP	Posterior Sagittal Anorectoplasty
PSARPVUP	Posterior Sagittal Anorectal Vaginal Urethral Plasty
REDCap	Research Electronic Data Capture
TP	Transanal Pull-Through
